# Intermediate outcomes following arch reconstruction with frozen elephant trunk, a single centre study

**DOI:** 10.1186/s13019-023-02140-5

**Published:** 2023-01-22

**Authors:** Therese Schagerholm Dahl, Rickard P. F. Lindblom

**Affiliations:** 1 Department of Surgical Sciences, Section of Thoracic Surgery, Uppsala University, Uppsala, Sweden; 2 Department of Cardiothoracic Surgery and Anesthesia, Uppsala University Hospital, 751 85 Uppsala, Sweden

**Keywords:** Frozen elephant trunk, Neurological outcomes, Aortic arch

## Abstract

**Background:**

Surgery on the aortic arch and proximal descending thoracic aorta can be lifesaving but is also associated with significant morbidity, ranging from minor infections to severe neurological impairments as well as a substantial risk of mortality. The aim of this study is to clinically assess outcomes, with special regards to neurologic injury, as well as to seek to identify predictors of in-hospital mortality in two patient groups with different underlying aortic pathology, aneurysms and dissections, undergoing arch/descending aortic repair.

**Methods:**

34 patients (17 aneurysms, 17 dissections) underwent surgery involving the arch and/or descending aorta, using the Thoraflex or E-Vita frozen elephant trunk graft. 40% were female. Subgroup analysis of aneurysms compared to dissections were performed. Mean follow-up time was 53.9 months and mean age 63.5 years.

**Results:**

In-hospital mortality was 18%. Survival was comparable between aneurysms and dissections. Incidence of spinal cord injury was 9% and stroke 9%. 67% suffered any form of neurological affection, when also cognitive afflictions were included. Perioperative reoperation rate was 29% (bleeding 21%, visceral ischemia 6%, infection 2%), the need for postoperative dialysis was 11% and a series of other minor complications such as atrial fibrillation and pleurocentesis were common.

**Conclusion:**

Postoperative dialysis was found to be a predictor of in-hospital mortality, while both dialysis as well as reoperation due to bleeding and/or visceral ischemia increased the risk for overall mortality, irrespective of preoperative diagnosis. Previous or current smoking appeared to be associated with negative outcomes regarding both in-hospital and overall mortality during follow-up.

*Trial registration* Retrospectively enrolled.

## Introduction

Even though surgery on the diseased thoracic aorta can be lifesaving, it is also vitiated with significant perioperative risks. Operations involving the aortic arch and proximal descending thoracic aorta can be especially challenging. Apart from perioperative mortality, other complications include those of the central nervous system (CNS) such as transient or permanent focal brain injury in the form of stroke, delirium, cognitive decline and ischemic spinal cord injury (SCI). Out of these, perhaps SCI is the most feared, as SCI has little potential of recovery [1].

The three supraaortic branches supply blood flow to the brain as well as the spinal cord, with the latter also receiving part of its arterial blood from smaller branches arising from the thoracoabdominal aorta. Hence, surgery on this part of the aorta confers risk of disrupting spinal cord blood flow.

Different adjunct strategies can be undertaken in order to minimize neurological injury. For example, moderate to deep hypothermia for CNS protection, selective antegrade cerebral perfusion (SACP) to maintain cerebral blood flow perioperatively, usage of spinal drain to monitor spinal perfusion pressure and regulating CSF drainage, as well as preoperative left subclavian artery (LSA) deviation are all suggested to protect against the development of SCI or stroke [2–7].

Surgical reconstruction of the arch/proximal descending aorta is increasingly performed using the frozen elephant trunk (FET) technique. The technique has several advantages, as it both enables a reconstruction/stabilization of the proximal descending aorta through a sternotomy in a one-stage procedure, and also creates a stable landing zone facilitating endovascular distal elongation in the aorta should this be required [8, 9]. In the current study, arch replacement was performed in two principally different patient groups; aneurysms of the ascending/arch/descending aorta or chronic type A or B dissections. All patients required complex repair which was performed using the FET technique with either the Thoraflex Hybrid or E-Vita grafts. The aim of the study was to assess clinical outcomes, with special regards to neurologic injury, as well as to seek to identify predictors of in-hospital mortality in this challenging group of patients.

## Material and methods

### Ethical permit

The study was approved by the local ethical committee in Uppsala under permit number 2012/296.

### Patients

All patients with complex aortic pathology, i.e. involving the arch and/or descending aorta scheduled to undergo surgery using the FET technique at the Department of Cardiothoracic Surgery and Anesthesia at Uppsala University Hospital who consented to having a spinal drain were after informed written consent intended for study inclusion. During the study period 49 patients were intended to treat for complex aortic pathology. 15 patients were excluded from the current study due to one or more of the following reasons: acute rather than chronic dissection, other operative strategy chosen (conventional ET, hemi-arch combined with TEVAR, open descending surgery, open and shut/exploratory procedure, isolated hemi-arch.) Due to the heterogeneity of the population a decision to include only patients operated with the FET technique on chronic entities were included in the final analysis. Thus, outcome among 34 patients was collected for statistical analysis. Patients were included during a period of 103 months, between May 2012 and December 2020. Clinical follow-up ended in March 2021 making the duration of the study 106 months with a mean follow-up time of 53.9 months.

### Endpoints

The primary endpoint was the mortality analysis in the group as a whole and secondary endpoints included morbidity in the cohort as a whole, with special attention to the neurologic afflictions.

### Surgery

All surgery was performed using the FET technique with extra-corporeal circulation (ECC). Sizing of graft dimensions and length of the frozen graft section was based on preoperative CT imaging after multidisciplinary discussion with specialized radiologists and vascular surgeons. Spinal drains were as a part of surgical routine placed preoperatively, unless contraindications were present or attempts were unsuccessful. The right subclavian artery was the preferred site of cannulation with addition of a separate cannula in the left carotid artery during SACP. Bilateral SACP is our standard practice and was achieved in close to all patients. Near infrared spectroscopy (NIRS) monitoring of cerebral perfusion was always performed. Moderate hypothermia of around 25 degrees Celsius was achieved by systemic cooling, after which hypothermic circulatory arrest (HCA) was initiated along with administration of cardioplegia and transection of the aorta. SACP (10 mL/kg/min, 18–20 degrees Celsius) was used when the hybrid stent graft was deployed into the descending aorta, after positioning of the first unstented portion in the arch was completed. The default approach was to anastomose the woven part of the graft in zone 0, i.e. to the distal ascending aorta just proximal of the innominate artery and the sewing cuff marking the start of the stentgraft portion was anastomosed in zone 2–3, i.e. mid-distal part of the arch remnant. When the two aortic anastomoses were performed, the aortic cross clamps were removed, circulation was restarted to the lower body and the arch vessels anastomosed to the branches of the hybrid graft in the case of the Thoraflex graft and *en-bloc* in the E-Vita group. The general practice was to opt for the shorter Thoraflex (100 mm stented section) prosthesis. The patient was rewarmed and eventually weaned of the ECC.

### Clinical data

All clinical data was collected retrospectively through reviews of the medical records.

Patient characteristics collected preoperatively were sex, age, BMI, comorbidities in the form of hypertension, hyperlipidemia, diabetes, COPD (chronic pulmonary obstructive disease) as well as a history of smoking, LVEF (left ventricular ejection fraction), grade of heart failure according to NYHA (New York Heart Association) Functional Classification, kidney function and previous heart surgery with opened pericardium. Kidney function was assessed by calculating absolute GFR based on the Lund-Malmö formula and then classified according to CKD (chronic kidney disease stage) [10]. LVEF was collected from the most recent echocardiogram.

Perioperative data included subdivisions of underlying diagnosis, preoperative subclavian deviation, spinal drain, concurrent heart valve surgery or coronary artery bypass grafting (CABG), maximum diameter of aneurysm, duration of ECC and aortic cross-clamping.

Lastly, postoperative variables were in-hospital mortality, neurological sequelae including paraparesis or paraplegia, severe confusion or delirium, hallucinations and stroke, as well as post lumbar puncture headache. Frequencies of reoperation were measured for bleeding, cardiac tamponade and infection. Occurrence of postoperative atrial fibrillation (POAF) with day of debut was recorded, as well as need for pleural drainage or pericardiocentesis. Additionally, incidence of acute renal failure requiring dialysis was registered. Vascular reintervention during the follow-up postoperative phase was included for analysis and was defined as stenting of the thoracoabdominal aorta by TEVAR and/or FEVAR.

### Biochemistry

Preoperative and postoperative day one and two serum values were collected for NT-proBNP (N-terminal prohormone brain natriuretic peptide), CKMB (creatine kinase myoglobin binding), ASAT (aspartate aminotransferase) and Troponin I. As NT-proBNP values, as well as preoperative Troponin I, were often missing and therefore excluded in the final model.

### Statistics

Descriptive statistics for a number of clinically relevant outcomes are reported in the results section. To investigate key predictors of clinical outcomes (most importantly neurological injury as well as in-hospital or subsequent mortality), these outcomes were also subjected to multivariate analysis using ordinary least squares regression (OLS) models. For binary outcomes, linear probability models (LPM) were chosen over logit or probit models due to simplicity of interpretation of coefficients, as they can be seen as simple changes in absolute probabilities. While this results in an unbounded prediction range, it was not a problem for this analysis since no point predictions were produced. Rather, the focus was on assessing the magnitude of various risk factors. Model specifications (i.e. the main independent and control variables for each model) have been laid out in detail in Tables 5, 6, 7, 8, 9, where each column represents one regression model and each row a single variable.

Diagnosis A is a binary variable indicating aneurysm as underlying diagnosis, as opposed to dissection. Other binary variables were kidney failure, heart failure, diabetes, smoking, reoperation, dialysis, concomitant valvular surgery, pleuro/pericardiocentesis, preoperative subclavian deviation and spinal drain. ECC and cross clamping times were measured in minutes. Serum levels of CKMB (µg/L) and ASAT (µkat/L) were measured.

## Results

### Preoperative data

Preoperative parameters are presented in detail in Table 1. Mean age was 64.0 ± 7.7 years in the cohort, but the age span varied from 26 to 77 years. Aneurysm patients were slightly older with a mean age of 68.0 ± 4.1 years, compared to dissection patients with mean age 60.0 ± 8.4 years.

**Table 1:** Preoperative data

		Aneurysm (n = 17)			Dissection (n = 17)				Total (n = 34)			
		n ($\underline{x}$)	% (SD)		n ($\underline{x}$)		% (SD)	*p* value for diff	n ($\underline{x}$)		% (SD)	
Mean age (SD), years		(68.0)	(4.09)		(60.0)		(8.44)	*.001*	(64.0)		(7.69)	
Sex												
	Female	11	64.7		3		17.6	*.003*	14		41.2	
	Male	6	35.3		14		82.4	*.003*	20		58.8	
Mean BMI (SD)		(25.3)	(3.49)		(27.6)		(3.48)	.058	(26.5)		(3.64)	
Hypertension		16	94.1		17		100	.311	33		97.1	
Hyperlipidemia		8	47.1		6		35.3	.489	14		41.2	
Diabetes		3	17.6		2		11.8	.627	5		14.7	
COPD		7	41.2		5		29.4	.477	12		35.3	
Smoking												
	Current	3	17.6		2		11.8	.627	5		14.7	
	Previous	7	41.2		9		52.9	.496	16		47.1	
	Never	7	41.2		6		35.3	.723	13		38.2	
EF												
	> 50	16	94.1		14		82.4	.288	30		88.2	
	40–49	1	5.88		3		17.6	.288	4		11.8	
	30–39	0	0		0		0	1	0		0	
	< 30	0	0		0		0	1	0		0	
Class of heart failure												
	NYHA I	4	23.5		4		22.2	1	8		23.5	
	NYHA II	6	35.3		5		27.8	.714	11		32.4	
	NYHA IIIa	5	29.4		4		22.2	.699	9		26.5	
	NYHA IIIb	1	5.88		3		16.7	.288	4		11.8	
	N/A	1	5.88		1		5.88	1	2		5.88	
Previous cardiac surgery		2	11.8		16		94.1	*.000*	18		52.9	
Renal function, CKD stage and absolute GFR (Lund-Malmö)												
	1 Normal (> 90)	1		5.88		6	35.3	*.029*	7	20.6		
	2 Mild (60–89)	9		52.9		6	35.3	.302	15	44.1		
	3 Moderate (30–59)	7		41.2		4	23.5	.275	11	32.4		
	4 Severe (15–29)	0		0		1	5.88	1	1	2.94		
	5 End stage (< 15)	0		0		0	0	1	0	0		

Italics indicates the significant values

Fourteen patients (41.2%) were female; eleven with aneurysms and three with dissections. Males were overrepresented among dissection patients (14 out of 17 patients).

All but one patient had hypertension (97.1%). Twenty-one patients (61.8%) were current or previous smokers and 12 (35.3%) fulfilled the criteria of COPD. Five patients (14.7%) had diabetes. Eighteen patients (52.9%) had previously undergone cardiac surgery, only two of which had aneurysm as underlying diagnosis.

Most patients had preoperative LVEF of > 50% (30 patients, 88.2%), and four patients (11.8%) had LVEF between 40 and 49%. The majority of the patients had normal or mild to moderate kidney failure.

### Perioperative data

Details of the perioperative data is presented in Tables 2 and 3. There was an even distribution between the two patient groups in regard to underlying diagnosis. Seventeen patients (50%) were diagnosed with aneurysm of the thoracic aorta and another seventeen patients (50%) had any of the three types of dissections mentioned above. The majority of the dissection patients had chronic type A dissections, only one was chronic type B.

**Table 2:** Diagnoses

Diagnosis	n	%
Dissections	17	50.0
- Chronic type A dissection	16	47.1
- Chronic type B dissection	1	2.94
Aneurysms	17	50.0

**Table 3:** Perioperative data

	Aneurysm (n = 17)		Dissection (n = 17)			Total (n = 34)	
	n ($\underline{x}$)	% (SD)	n ($\underline{x}$)	% (SD)	*p* Value for diff	n ($\underline{x}$)	% (SD)
Type of graft							
Thoraflex	14	82.4	12	70.6	*.418*	26	76.5
Evita graft	3	17.6	5	29.4	*.418*	8	23.5
Preoperative subclavian deviation	7	41.2	5	29.4	*.477*	12	35.3
Spinal drain	14	82.4	13	76.4	*.677*	27	79.4
Heart valve surgery	3	17.6	7	41.2	*.129*	10	29.4
CABG	1	5.88	1	5.88	*1*	2	5.88
Mean maximum diameter of aneurysm (SD), mm	(64.3)	(8.11)	(62.4)	(12.8)	*.607*	(63.4)	(10.6)
ECC-time							
Mean duration (SD), min	(270)	(46.3)	(352)	(93.5)	*.003*	(311)	(83.9)
Mean aortic cross clamp time (SD), min	(126)	(36.5)	(168)	(55.3)	*.014*	(147)	(50.8)

### Postoperative data

Postoperative outcome is presented in Table 4. Clinical follow-up lasted between 3 and 106 months, with a mean follow up time of 53.9 months (range 3–103).

**Table 4:** Postoperative data

Diagnosis	Aneurysm (n = 17)		Dissection (n = 17)			Total (n = 34)	
	n ($\underline{x}$)	%	n ($\underline{x}$)	%	*p* Value for diff	n ($\underline{x}$)	%
Mortality, total	7	41.2	5	29.4	*.477*	12	35.3
In-hospital	3	17.6	3	17.6	*1*	6	17.6
Within follow-up	4	23.5	2	11.8	*.370*	6	17.6
Neurological sequelae	13	76.5	10	58.8	*.271*	23	67.6
Paraparesis	2	11.8	0	0	*.141*	2	5.88
Paraplegia	0	0	1	5.88	*.311*	1	2.94
*Severe confusion or delirium	8	47.1	8	47.1	*1*	16	47.1
*Hallucinations	8	47.1	8	47.1	*1*	16	47.1
*Both	5	29.4	8	47.1	*.293*	13	38.2
Stroke	1	5.88	2	11.8	*.546*	3	8.82
Reoperation	7	41.2	3	17.6	*.129*	10	29.4
Bleeding	2	11.8	3	17.6	*.627*	5	14.7
Tamponade	2	11.8	0	0	*.141*	2	5.88
Visceral ischemia	2	11.8	0	0	*.141*	2	5.88
Infection	1	5.88	0	0	*.311*	1	2.94
Pleural drainage	4	23.5	3	17.6	*.670*	7	20.6
Pericardiocentesis	2	11.8	0	0	*.141*	2	5.88
Dialysis	3	17.6	1	5.88	*.288*	4	11.8
Atrial fibrillation (AF)	11	69.2	10	58.8	*.279*	21	61.8
- Post-op debut (AF), day	(2.36)	(1.80)	(4.80)	(2.25)	*.001*	(3.52)	(2.34)
Post lumbar puncture headache	2	11.8	3	17.6	*.627*	5	14.7
Postoperative vascular reintervention, total	7	41.2	12	70.6	*.080*	19	55.9
≥ 2 vascular reinterventions	1	5.88	7	41.2	*.012*	8	23.5

### Mortality

#### In-hospital mortality

There were six (17.6%) in-hospital mortalities in the entire cohort, four of which were female. All occurred within 3–30 days postoperatively. Two patients died due to visceral ischemia, one from aortic rupture, one from cardiac failure and the remaining two from MOF.

#### Mortality within follow-up

Another six patients (17.6%), two of which were female, died during follow-up, rendering a total mortality of 12 patients (35.3%) within the study period. The causes of death during follow-up among two of the patients were due to MOF (multiorgan failures) following graft infection, 8.5 and 17 months postoperatively. Another patient died 45 months postoperatively due to MOF following several attempts at reintervention in the thoracoabdominal aorta. The last three patients included one death from aortic rupture, one from cardiac failure and one unknown.

### Morbidity

A total of 24 patients (67.6%) suffered some form of neurologic injury. SCI was seen in three (8.8%), where two patients had paraparesis and the remaining patient paraplegia. The patients with paraparesis both had aneurysms as underlying diagnosis, while the patient with paraplegia was a previous type-A aortic dissection.

One patient had an aneurysm involving the arch as well as the ascending aorta. ECC time was 327 min (mean 270 ± 46.3 min aneurysm group) and aortic cross clamp time 153 min (mean 126 ± 36.5 min aneurysm group). The patient did not undergo preoperative deviation of the subclavian artery, but reimplantation was made intraoperatively. Attempts at spinal drain were made but unsuccessful.

The other paraparetic patient also had an aneurysm of the ascending aorta and the arch. ECC was 229 min and cross clamp time 142 min. The patient underwent preoperative subclavian deviation. Attempts at establishing a spinal drain were unsuccessful.

The patient with chronic type A dissection had an ECC time of 523 min, cross clamping time of 303 min, spinal drain was established and LSA was reimplanted perioperatively.

Three patients (8.8%) suffered a stroke with residual hemiplegia, one of which had an extensive aneurysm localized to the ascending, arch and the descending aorta. The patient had been deviated preoperatively and received a spinal drain before undergoing surgery with FET procedure. The remaining two was one chronic type-A dissection with no spinal drain and intraoperatively reimplanted LSA and one type B-chronic dissection who received a spinal drain and intraoperative reimplantation of the LSA.

Severe confusion/delirium occurred in 16 patients (47.1%) and 16 patients (47.1%) experienced hallucinations. 13 of these patients (38.2%) had both delirium and hallucinations.

POAF occurred in 61.8% of the patient population but did not seem coupled to any other serious complications. Other minor complications included seven patients (20.6%) requiring pleural drainage and two (5.9%) pericardiocentesis. Four patients (11.8%) had acute renal failure requiring dialysis. All were female.

In the aneurysm group, seven patients (41.2%) were reoperated on during the immediate postoperative phase; two for bleeding, two for cardiac tamponade secondary to bleeding, two for visceral ischemia and one for infection (deep sternal wound infection, not involving the graft). In the dissection group three patients (17.6%) were reoperated, all due to bleeding.

### Reintervention during follow-up

Nineteen patients (55.9%), twelve (70.6%) with dissections and seven (41.2%) with aneurysms, underwent postoperative reintervention and/or vascular surgery in the form of either TEVAR or FEVAR. The majority (fourteen of nineteen, 74%) of the reinterventions were elective extensions, five (26%) were urgent/emergent. Eight (23.5%) of the patients, seven of which were dissection patients, had more than one reintervention done, one of which was urgent.

### Attempts at defining predictive factors

Additional statistical analysis with regression models were used to assess possible prognostic factors for mortality, both overall and in-hospital, neurologic injury and specific focus on SCI, as well as POAF.

When analyzing preoperative parameters age, sex, underlying diagnosis, kidney failure, diabetes, smoking, heart failure and BMI, being a previous or current smoker was associated with an increased risk for overall mortality by 37.0 percentage points (*p* < 0.1) (Tables 5 and 6). When correcting for age, sex and diagnosis for peri- and postoperative variables ECC and aortic cross clamping time, reoperation for bleeding and/or visceral ischemia, patients with acute renal failure requiring dialysis during postoperative ICU treatment had a 51.9 percentage point (*p* < 0.05) increase in risk for in-hospital mortality (Table 5).

**Table 5:** In-hospital mortality

Variables	(1)	(2)
	In. hosp. mort	In. hosp. mort
Age	0.00572	0.0151
	(0.0130)	(0.00925)
Female	0.186	0.0634
	(0.155)	(0.151)
Diagnosis A	− 0.141	− 0.147
	(0.174)	(0.168)
Kidney failure	0.240	
	(0.222)	
Comorbid diabetes	0.0212	
	(0.203)	
Smoking	0.219	
	(0.138)	
Heart failure	− 0.0467	
	(0.0629)	
BMI	0.0257	
	(0.0200)	
ECC time		0.000624
		(0.00108)
Cross clamp time		0.000780
		(0.00171)
Reoperation		0.0815
		(0.142)
Dialysis		0.519**
		(0.216)
Constant	− 1.110	− 1.134*
	(0.784)	(0.633)
Observations	34	34
R-squared	0.322	0.410

Standard errors in parentheses

***p < 0.01, **p < 0.05, *p < 0.1

Each column represents a single multiple regression model with in-hospital mortality as the outcome and the variable listed in each row as a covariate. For binary covariates, yes = 1 and no = 0, meaning that if the variable is present, the resulting coefficient is the absolute increase in risk, expressed as a fraction. For non-binary covariates, the resulting coefficient is the absolute increase in risk, expressed as a fraction, per one unit increase. Non-binary values are measured as follows; age (years), BMI (kg/m^2^), ECC and cross clamping time (minutes). Diagnosis A: aneurysm

**Table 6:** Overall mortality

Variables	(1)	(2)
	Overall mort	Overall mort
Age	− 0.00747	0.00677
	(0.0171)	(0.0107)
Female	0.111	0.00665
	(0.203)	(0.174)
Diagnosis A	0.00530	− 0.0538
	(0.229)	(0.195)
Kidney failure	0.298	
	(0.292)	
Comorbid diabetes	− 0.0667	
	(0.267)	
Smoking	0.370*	
	(0.181)	
Heart failure	− 0.00114	
	(0.0827)	
BMI	0.00124	
	(0.0263)	
ECC time		9.59e−05
		(0.00124)
Cross clamp time		0.00238
		(0.00197)
Reoperation		0.435**
		(0.164)
Dialysis		0.513**
		(0.249)
Constant	0.290	− 0.654
	(1.029)	(0.732)
Observations	34	34
R-squared	0.224	0.477

Standard errors in parentheses

***p < 0.01, **p < 0.05, *p < 0.1

Each column represents a single multiple regression model with overall mortality as the outcome and the variable listed in each row as a covariate. For binary covariates, yes = 1 and no = 0, meaning that if the variable is present, the resulting coefficient is the absolute increase in risk, expressed as a fraction. For non-binary covariates, the resulting coefficient is the absolute increase in risk, expressed as a fraction, per one unit increase. Non-binary values are measured as follows; age (years), BMI (kg/m^2^), ECC and cross clamping time (minutes). Diagnosis A: aneurysm

The association between in-hospital mortality and dialysis did not show marked differences between the A/D groups, indicating that it may be an important prognostic factor during the immediate postoperative phase after complex aortic surgery with the FET procedure in itself, regardless of underlying diagnosis (Table 5).

Female gender increased the risk of requiring postoperative dialysis by 23.7 percentage points (*p* < 0.1) and similarly, reoperation during the immediate postoperative phase also increased that risk by 23.5 percentage points (*p* < 0.1) when correcting for age, any stage of preoperative kidney failure, diabetes, underlying diagnosis as well as ECC and cross clamping times (Table 7).

**Table 7:** Dialysis

Variables	(1)	(2)
	Dialysis	Dialysis
Age	0.00547	0.00662
	(0.00741)	(0.0110)
Female	0.296**	0.237*
	(0.116)	(0.131)
ECC time	0.000337	0.000236
	(0.000904)	(0.00105)
Cross clamp time	0.000801	0.000659
	(0.00155)	(0.00165)
Kidney failure		0.0968
		(0.181)
Diagnosis A		− 0.0754
		(0.154)
Comorbid diabetes		− 0.143
		(0.169)
Reoperation		0.235*
		(0.123)
Constant	− 0.577	− 0.662
	(0.548)	(0.632)
Observations	34	34
R-squared	0.232	0.343

Standard errors in parentheses

***p < 0.01, **p < 0.05, *p < 0.1

Each column represents a single multiple regression model with dialysis as the outcome and the variable listed in each row as a covariate. For binary covariates, yes = 1 and no = 0, meaning that if the variable is present, the resulting coefficient is the absolute increase in risk, expressed as a fraction. For non-binary covariates, the resulting coefficient is the absolute increase in risk, expressed as a fraction, per one unit increase. Non-binary values are measured as follows; age (years), BMI (kg/m^2^), ECC and cross clamping time (minutes). Diagnosis A: aneurysm

Neither analysis of either the A/D group separately, nor the entire cohort, could show diagnosis, age, duration of ECC and aortic cross-clamping, previous cardiac surgery or preoperative kidney failure as predictors of in-hospital mortality (Table 5).

Risk for overall mortality increased by 43.5 percentage points (*p* < 0.05) when undergoing reoperation for bleeding and/or visceral ischemia and by 51.3 percentage points (*p* < 0.05) when postoperative kidney failure requiring dialysis occurred. This held true after correcting for diagnosis, age, sex, as well as duration of ECC and aortic cross-clamping (Table 6). In contrast, no risk factor for reoperation could be found when analyzing parameters underlying diagnosis, age, sex, duration of ECC and aortic cross clamping, diabetes or preoperative kidney failure (Table 8).

**Table 8:** Reoperation, POAF, neurological complications

Variables	(1)	(2)	(3)
	Reop	A-fib	Neuro
Age	− 0.00953	0.00390	0.0211
	(0.0127)	(0.0145)	(0.0130)
Female	0.177	− 0.00390	− 0.363*
	(0.195)	(0.212)	(0.204)
ECC time	− 0.000428		− 0.000986
	(0.00151)		(0.00152)
Cross clamp time	0.000886		0.000967
	(0.00240)		(0.00243)
Diagnosis A	0.230	0.0688	0.138
	(0.233)	(0.251)	(0.241)
Concomit valvular surgery		0.150	
		(0.219)	
Pleuro/pericardiocentesis		− 0.0351	
		(0.232)	
Preop subclavian deviation			0.102
			(0.179)
Spinal drain			− 0.218
			(0.212)
Constant	0.719	0.299	− 0.292
	(0.856)	(0.882)	(0.870)
Observations	34	34	34
R-squared	0.111	0.025	0.237

Standard errors in parentheses

***p < 0.01, **p < 0.05, *p < 0.1

Each column represents a multiple regression model with the following outcomes: Column 1: reoperation. Column 2: Atrial fibrillation. Column 3: Neurological sequelae. Each row contains a covariate in the model. For binary covariates, yes = 1 and no = 0, meaning that if the variable is present, the resulting coefficient is the absolute increase in risk, expressed as a fraction. For non-binary covariates, the resulting coefficient is the absolute increase in risk, expressed as a fraction, per one unit increase. Non-binary values are measured as follows; age (years), ECC and cross clamping time (minutes). Diagnosis A: aneurysm

Risk for any type of neurological injury (SCI, stroke, delirium and hallucinations) in the cohort increased by 36.3 percentage points (*p* < 0.1) if the patient was male, when correcting for variables age, underlying diagnosis, duration of ECC and aortic cross-clamping, preoperative left subclavian deviation and spinal drain. Neither preoperative deviation of LSA or spinal drains appeared to be protective against neurologic injury—just over 35% the patients with postoperative sequalae underwent preoperative subclavian deviation. Among the 22 patients who did not, frequency of neurologic injury was 64% (Table 8).

No parameters could be determined to serve as predictors of POAF, when accounting for concomitant valvular surgery, pleural or pericardial effusion requiring drainage, underlying diagnosis, age and sex.

### Biochemistry

Serum levels for CKMB, Troponin I and ASAT were collected and analyzed as potential predictors for mortality during the immediate postoperative phase (Table 9). Preoperative Troponin I was only available for two patients and was therefore excluded in the final model. ASAT and CKMB levels were compared as delta values preoperatively as well as POD1 (delta 1) and POD2 (delta 2) in order to evaluate increases or decreases as potential markers for in-hospital mortality. Statistical analysis indicated that increases in all delta values correlated to in-hospital mortality, but although the findings were statistically significant (*p* < 0.05), percentage points were only increased by relatively low values for a 1.0 unit increase in delta levels; delta CKMB 1 by 2.35 pp (*p* < 0.01), delta CKMB 2 by 2.05 pp (*p* < 0.05), delta ASAT 1 4.20 (*p* < 0.01), delta ASAT 2 by 0.577 pp (*p* < 0.01).

**Table 9:** Cardiac biomarkers

Variables	(1)	(2)
	In. hosp. mort	In. hosp. mort
delta_ASAT1	0.0420***	
	(0.0148)	
delta_ASAT2	0.00577***	
	(0.00208)	
delta_CKMB1		0.0235***
		(0.00738)
delta_CKMB2		0.0205**
		(0.00907)
Constant	0.0593	− 0.0356
	(0.0654)	(0.149)
Observations	34	27
R-squared	0.332	0.302

Standard errors in parentheses

***p < 0.01, **p < 0.05, *p < 0.1

Each column represents a single multiple regression model with in-hospital mortality as the outcome and the variable listed in each row as a covariate. The covariates are measured as changes in ASAT or CKMB between the pre-operative period and POD1, or between POD1 and POD2. Units for the covariates are µg/L (CKMB) and µkat/L (ASAT)

## Discussion

The current study evaluates mid-term outcomes following FET surgery for either dissection or aneurysm, performed at a single centre, Uppsala University Hospital. The incidence of in-hospital mortality is comparable but in the higher interval of the commonly reported levels (7.7–15.9%) (9–11), but the levels of neurological injury (stroke and SCI) are similar to previously reported results [4, 6–9, 11, 12].

Martens et al. determined postoperative dialysis and reoperation for bleeding as risk factors for mortality after total aortic arch repair, both for ET and FET procedures [13].

In a recent study, Mkalaluh et al. found similar results indicating duration of surgery, postoperative bleeding requiring surgical exploration and acute renal failure requiring dialysis as predictors of in-hospital mortality. For overall mortality, acute renal failure requiring dialysis was the sole predictor where statistical significance was observed [14].

In this study, patients who required dialysis postoperatively had a statistically significant increase in risk of both in-hospital mortality and overall mortality. Avoidance of renal failure is of essence in order to decrease mortality after FET surgery. This could perhaps be aided by better perioperative renal protection and perfusion and is an aspect that should be considered for further evaluation. Potentially increased awareness and more meticulous attention to visceral perfusion could decrease the renal injuries and perhaps also mortality.

Female gender as well as reoperation during the immediate postoperative period were the only predictors for requiring postoperative dialysis, either in the A/D groups or the entire cohort, but these findings did not have a strong statistical significance (*p* < 0.1). Regression modelling did not prove preoperative kidney failure to be associated with postoperative requirement for dialysis, illustrating that it likely is the *periprocedural* renal ischemia that causes the need for dialysis. Of interest is that neither ECC nor aortic cross-clamping time were correlated with the need for dialysis. This implies that it is perhaps rather the quality than the duration of perfusion that matters.

Beller et al. found that in-hospital mortality was twice as high for female patients compared to males when undergoing surgery for ascending aortic aneurysms [15]. Female aneurysm patients were also approximately five times more likely to require post-operative dialysis (*p* = 0.024) [15]. However, in the setting of dissections, the evidence is conflicting, older data suggest worse outcomes for women [16], whereas newer data proposes similar results between the genders [17]. In the current study there was also a gender difference within the groups, with more women in the aneurysm than in the dissection group. Although not statistically significant, male patients appeared to be more prone to postoperative neurological sequelae.

In contrast to previous findings, patients who were subject to reoperation due to bleeding and/or visceral ischemia had an increased risk of overall mortality, but for in-hospital mortality there was no evident correlation. Of course, we cannot rule out that re-exploration for bleeding was a surrogate marker for technically more challenging conditions. However, it emphasizes the critical need to avoid this complication in the current patient group—stringent attention to optimizing coagulation should be performed, and perhaps the operating surgeon should be offered optimal assistance in the sometimes extended and tiresome hemostatic phase of the operation.

One could expect visceral ischemia to be more of an issue in the dissection group as compared to the aneurysm group. However, we noted the opposite; there was no intestinal ischemia in the dissection group whereas there were two cases amongst the aneurysms that required urgent abdominal surgery. In both these cases, dislodgement of thrombi from the aneurysmal sac/aortic wall were the putative cause. Due to small numbers, this was not statistically significant. The finding is still interesting and it is worth bearing in mind that visceral malperfusion is an issue even in the aneurysm group.

In general, neurological afflictions are very common, if one also considers delirium and hallucination. Delirium among cardiac surgery patients have previously been reported to occur at around 25%, with a wide range from 10 to 80% depending on the type of surgery and the efficacy in registering [18]. Delirium is probably often underdiagnosed, and perhaps underrated as a complication as it often necessitates prolonged ICU stay.

Previous studies have detected aneurysmal pathology, diabetes, previous aortic surgery as risk factors of SCI [19]. In the current study, three patients developed postoperative SCI. The underlying diagnosis was aortic aneurysm in two of these patients, one of which had undergone previous aneurysmal surgery, and the third patient had a chronic type A dissection, but none had diabetes. Usage of spinal drain and pre-operative subclavian deviation did not statistically prove to be protective measures taken to minimize neurological damage. However, it is striking that two out of three patients that developed SCI did not have a spinal drain, compared to the general cohort where 80% had a spinal drain, and of course raises the suspicion that there could be a link. But considering the small sample size as well as the low number of permanent neurological injury suffered, no statistical conclusion can be drawn as to whether these efforts are rendered ineffective. However, we empirically advocate the use of these methods still strongly should be considered for patients undergoing this type of complex surgery, as proposed [6].

The possibility of finding predictors for SCI could increase with a larger cohort or through meta-analyses. However, definitions of both SCI and neurologic injury have previously not been subject to standardized classification, providing less reliability to reported results [9].

Apart from the above-mentioned serious complications, major aortic surgery leads to several minor complications that still require intervention, such as pleural and pericardial effusions and atrial fibrillation. These issues are readily handled at a cardiothoracic centre, but in units less accustomed to interventions and with less monitoring resources these could lead to serious adverse events. POAF occurred at a rate of 61.8%, which is higher than the commonly cited 15–45% frequency after cardiac surgery in general. However, this is not surprising given the volume shifts, electrolytical derangements and general inflammatory state after this type of major surgery why the comparison is perhaps not completely relevant. Concomitant valvular surgery, pleural and pericardial effusion as well as age have previously been recorded as risk factors for developing POAF but this could not be shown within our cohort [20–22]. Neither pre-operative NYHA class was predictive of POAF (data not shown).

Rate of vascular reintervention differed between the A/D groups, with higher rates among dissection patients. A recent study analyzed patient characteristics of aortic reinterventions after the FET procedure, where 33% of the patients underwent both anticipated and unexpected reintervention, with no difference regarding underlying diagnosis [23]. In our study, overall, half of the patient population received TEVAR and/or FEVAR. Almost a quarter of the patients required more than one reintervention, the majority of these were dissection patients. The hybrid FET procedure was developed in order to facilitate reintervention of this kind. The rates of reintervention in the current study are higher than often reported. A majority of these were planned reintervention. A hypothesis is that the use of the shorter Thoraflex prosthesis (100 mm frozen section) could entail a higher rate of elongation procedures, but hopefully with the benefit of less SCI. Further analysis of reintervention rate regarding patient characteristics would be of interest for further study, but is beyond the scope of the current study.

### Biomarkers

Although lab values and biomarkers have previously been studied as predictors for outcomes in patients undergoing complex aortic surgery, research is limited and potential to find prognostic factors should not be disregarded. A previous multicentre study found increased levels of preoperative serum creatinine, as well as postoperative dialysis, to be associated with in-hospital mortality [24]. In this study, creatinine levels preoperatively were analyzed to determine CKD stage by calculating absolute GFR. Although previous studies have reported preoperative kidney failure to be a risk factor for negative outcomes postoperatively, this could not be confirmed in this study. This is interesting, as it suggests that it is de novo/perioperatively acquired kidney injury that is linked to mortality.

A recent study analyzed both serum and CSF samples pre-, peri- and postoperatively to detect biomarkers for SCI and neurologic injury after aortic surgery. Albeit no statistically significant factor was found to predict SCI alone, biomarkers did differ between injured and non-injured, indicating this as an area where further investigation could aid in recognizing prediction factors for postoperative outcomes for SCI and neurologic injury [25].

Perioperative levels of CKMB, Troponin-I and ASAT were collected and analyzed as potential predictors for mortality during the immediate postoperative phase. Results indicated that increases in levels of both CKMB and ASAT could be used as a predictor for in-hospital mortality. Further analysis is indicated, preferably with a larger cohort, for determining whether these findings are caused by other underlying mechanisms that may increase the risk for in-hospital mortality, or if the values are predictors without causative effect.

Additionally, standardized procedures for pre- and postoperative lab testing could be used to better evaluate the potential use of serum values as predictors of in-hospital mortality.

### Limitations

Due to the limited size of the analyzed cohort, further studies with increased sample sizes would be a valuable means of possibly finding statistically significant prediction factors for surgical outcome.

## Conclusion

The current study is limited in size, and ultimately larger cohorts could identify additional important answers. However, some conclusions that contribute to the pool of knowledge regarding this complex group of patients can be drawn. Incidence of in-hospital mortality increased with postoperative acute renal failure requiring dialysis and female patients had a higher risk of developing the latter. Reoperation due to bleeding and/or visceral ischemia during the immediate postoperative phase was a predictor for dialysis and overall mortality. Male patients had a higher risk for neurologic injury. However, further study is needed to determine the importance of gender. Minor complications with the potential for worsening occur in practically all patients operated for complex aortic disease. Survival through the immediate perioperative phase confers a good mid-term survival, albeit with the need for reinterventions and more so in the dissection group. Lastly, solely based upon our experience, but not underpinned by robust data, we agree with the current recommendations of using spinal drains, and propose that preoperative deviation of the subclavian artery is considered if difficult intraoperative connection to the FET-graft could be anticipated. We emphasize that all measures to avoid re-exploration for bleeding and dialysis are taken and propose that it is likely not the duration but the quality of the perfusion that matters. In summary, these measures could potentially improve outcomes in this challenging patient population.

## Data Availability

Not applicable.

## Abbreviations

ECC: Extra-corporeal circulation
FET: Frozen elephant trunk
LSA: Left subclavian artery
POAF: Postoperative atrial fibrillation
SACP: Selective antegrade cerebral perfusion
SCI: Spinal cord injury
HCA: Hypothermic circulatory arrest
TEVAR/FEVAR: Thoracic/fenestrated endovascular aortic repair
NT-proBNP: N-terminal prohormone brain natriuretic peptide
ASAT: Aspartate aminotransferase
CKMB: Creatine kinase myoglobin binding
MOF: Multi organ failure
